# Effect of Caffeine Copigmentation of Anthocyanin Dyes on DSSC Efficiency

**DOI:** 10.3390/ma12172692

**Published:** 2019-08-22

**Authors:** Irén Juhász Junger, Suphawit Udomrungkhajornchai, Nils Grimmelsmann, Tomasz Blachowicz, Andrea Ehrmann

**Affiliations:** 1 Faculty of Engineering and Mathematics, Bielefeld University of Applied Sciences, 33619 Bielefeld, Germany; 2 Institute of Physics—CSE, Silesian University of Technology, 44-100 Gliwice, Poland

**Keywords:** caffeine, anthocyanin, copigmentation, dye, dye-sensitized solar cell (DSSC), photo-electrode

## Abstract

Caffeine is known to influence the absorbance spectrum of anthocyanin dyes. Such dyes are often used as sensitizers in dye-sensitized solar cells (DSSCs). Natural dyes, like anthocyanins, yield only small DSSC efficiencies, but are of high interest since they are usually non-toxic and inexpensive. Here we report on the influence of copigmentation of anthocyanins, taken from commercially available tea, with caffeine. In this way, the efficiencies were increased for measurements with a solar simulator as well as with ambient light. In addition, the well-known pH dependence of the efficiency of DSSCs dyed with anthocyanins was shifted—while a pH value of 1–2 was ideal for pure anthocyanins used as dyes, a higher pH value of 2–3 was sufficient to reach the maximum efficiencies for caffeine-copigmented dyes. This means that instead of reducing the pH value by adding an acid, adding caffeine can also be used to increase the efficiency of DSSCs prepared with anthocyanins. Finally, a comparison of several literature sources dealing with anthocyanin-based DSSCs allows for evaluation of our results with respect to the work of other groups.

## 1. Introduction

Dye-sensitized solar cells (DSSCs) have been investigated by diverse research groups since their first presentation in the scientific literature [1]. A large advantage of DSSCs, as opposed to common silicon-based solar cells, is the possibility to produce them from non-toxic, low-cost materials without a cleanroom. On the other hand, the highest conversion efficiencies are reached with very specialized materials, which typically lack these advantages [2].

Since no special conditions are needed for the preparation of DSSCs, they may be suitable for integration into textiles. Several research groups have made an effort in this direction [3,4,5]. During the integration of DSSCs into textiles, the use of toxic materials should be avoided, for example through a replacement of the widely used ruthenium based dyes by natural dyes. However, DSSCs with natural dyes are known to yield low energy conversion efficiencies. In order to eliminate this handicap, different natural dyes and dye sources were examined by many research groups. With leaf extracts, for example, efficiencies up to 0.011% were reached [6], while curcumin resulted in an efficiency of 0.33% [7]. Combining ternatin with chlorophyll led to efficiencies of up to 0.26% [8], and paprika resulted in an energy conversion efficiency of 0.14% [9]. For one of the most often used groups of dyes, the anthocyanins which can be found in a broad variety of flowers and berries, efficiencies between 0.03% and 0.15% for artificial sunlight [10,11,12,13,14] and between 0.06% and 0.33% for different indoor illumination situations were found [15]. 

The achieved efficiencies are too low for a commercial use. One possibility to increase the efficiency of DSSCs based on natural dyes is co-sensitizing, i.e., mixing two or more different dyes. While mixing multiple natural dyes indicates a slight increase of efficiencies [16,17,18], adding a toxic and expensive ruthenium-based dye like the well-known N719 to natural dyes, extracted from plants [19], leads to a higher efficiency increase; however, the efficiency is smaller than with pure N719.

Our aim is to develop a textile-based DSSC. For that purpose, it is important to find a natural dye with good absorption properties. In a previous work, we have investigated DSSCs dyed with anthocyanins and found a significant efficiency improvement when the pH value of the dye extract was decreased to 1.1 [12]. However, many textiles cannot withstand a treatment with strongly acidic medium. Therefore, we are looking for a way to increase the efficiency using dyes with a moderate acidic pH value.

In this study we investigated DSSCs dyed with anthocyanins co-pigmented by caffeine. Depending on the pH value, the anthocyanins can be found in different forms [20,21,22,23]. At pH = 1, only the red flavylium cation is present in the solution, while at higher pH values the dye solution contains a mixture of colored and colorless forms: the flavylium cations are partly deprotonated to the blue quinoidal bases and hydrated to the colorless hemiketals. At pH = 5 the hemiketals dominate in the solution, resulting in a significant loss of its color. Above pH = 7 the anthocyanins begin to degrade irreversibly into pale yellow chalcones [20,21,22,23]. In a previous work, we have shown that a decrease of the dye-solution pH value to 1.1 increases the energy-conversion efficiency of the DSSC by approximately a factor of two, although the highest dye adsorption on TiO_2_ was measured for pH = 3–4 [12]. This indicates that at pH = 3–4 the colorless hemiketals were also adsorbed on TiO_2_, and that an increase of the fraction of colored species in the dye solution for this pH region would further increase the efficiency. Since anthocyanins are present in red wine and are widely used as red colorants in the food industry, many efforts were made to stabilize their color in less acidic conditions over a longer period of time [21]. It was found that a copigmentation of anthocyanins with caffeine increases the concentrations of flavylium cations and quinoidal bases at moderate pH values, leading to a hyperchromic effect and a bathochromic shift of the absorption maximum of the dye solution [21,22,23]. As this effect increases the amount of colored species in the pH region of highest dye adsorption on TiO_2_, an increase of the DSSC efficiency with moderately acidic dyes is expected. Here, the impact of copigmentation of anthocyanin dyes by caffeine on the photovoltaic properties of DSSCs is investigated and, to our knowledge, reported for the first time.

## 2. Materials and Methods

Our final goal is to develop textile based DSSCs. However, this paper is focused on the investigation of the dye layer. Therefore, to avoid effects originating from possible irregularities of self-made textile based electrodes or self-made fluorine-doped tin oxide (FTO) and TiO_2_ coatings on glass, commercial FTO and TiO_2_ glasses (Man Solar, Petten, The Netherlands) were used for preparation of DSSCs. In addition, using commercially available glasses, similar to most other groups who are not investigating the TiO_2_ layer, enables comparison of the component under investigation. The thickness of the TiO_2_ layer was approx. 1 µm.

**Figure 1:**
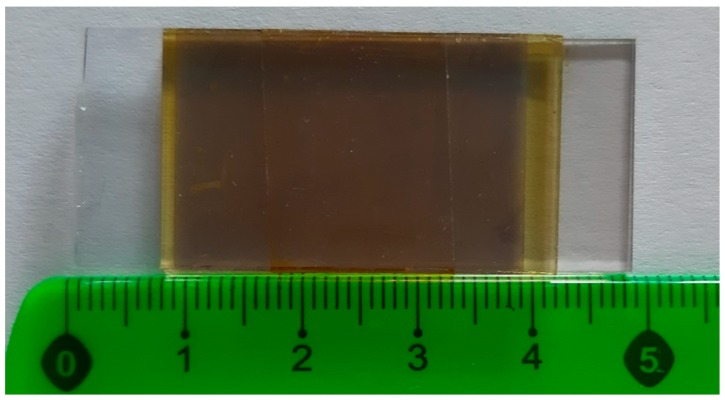
DSSC prepared in this study.

The counter electrodes were prepared by coating of the FTO glasses with a copy pencil, while the working electrodes were prepared as follows. For the dye, 10 g forest fruit tea (Mayfair) were ground in a mortar and dispersed in 500 mL of distilled water at 28 °C for 30 min before the solution was filtered. This original dye solution has a pH value of 2.89, as measured with a Star A329 pH meter (Fisher Scientific GmbH, Schwerte, Germany). Parts of the solution were modified with HCl or NaOH, respectively, to reach pH values of 1.00, 2.00, 3.00, 4.00, and 5.00. Half of each pH-modified and original solution was further modified by adding 0.2 g caffeine (min. 98.5%, anhydrous, purchased from Carl Roth, Karlsruhe, Germany) to 30 mL dye solution. After 135 min, the TiO_2_ coated glasses were placed in the dye solution for 48 h before the excess dye was rinsed with water, and the front electrodes were dried at the air. The electrodes were put together, fixed by an adhesive tape, and an electrolyte (type 016, man Solar, Petten, The Netherlands) was filled into the gap between the electrodes. The active area of the cells was 6 cm^2^. Three specimens of each cell type were prepared and the results were averaged. Photographic images and sketches of typical DSSCs prepared in this way can be found, e.g., in Ref. [24], while a photograph of one of the recent cells is depicted in Figure 1.

The electrical characterization of the DSSCs was performed by a Keithley 2450 sourcemeter (Keithley Instruments GmbH, Germering, Germany). The samples were illuminated with a daylight lamp (halogen lamp with 3000 K color temperature) or a solar simulator LS0500 (LOT-Quantum Design GmbH, Darmstadt, Germany, with AM 1.5 G spectrum), respectively, applying 100 mW/cm² in each experiment. In this way, the DSSC performance under indoor and outdoor illumination conditions was tested, since DSSCs are known to function well in indoor conditions [2], and future textile-based solar cells could be integrated into garments and would thus be worn inside and outside buildings.

For the measurement of ultraviolet/visible (UV/Vis) absorption spectra, the equilibrated dye solutions were diluted by distilled water by a factor of 5. The measurements were carried out by a spectrophotometer Genesys 10S (Thermo Fisher Scientific, Waltham, MA, USA).

## 3. Results and Discussion

As mentioned in the introduction, in a previous work, where we have studied the influence of the pH value of the anthocyanin dye solution on the energy-conversion efficiency of DSSCs, the highest efficiency was found for pH = 1.1, in spite of the fact that the dye adsorption on TiO_2_ layer was most intensive between pH = 3 and pH = 4 [12]. This mismatching can be explained as follows. It is known that the anthocyanin molecules undergo structural changes by varying pH value of the solution. For example, at pH = 1 almost all anthocyanin molecules are in the form of flavylium cations, which have an intensive red color with a maximum in the absorption spectrum at the wavelength of 520 nm. At higher pH values, the flavylium cations are partly hydrated to colorless hemiketals and partly deprotonated to blue quinoidal bases, and a fraction of anthocyanins is degraded to pale yellow chalcones. At all pH values, the dye solution contains a mixture of all species, whose molar fractions vary with varying pH value. As mentioned above, at pH = 1 the flavylium cations are dominant in the solution. For increasing pH, the fraction of hemiketals is increasing at the expense of colored species, which results in fading of the dye color [21,22,23].

**Figure 2:**
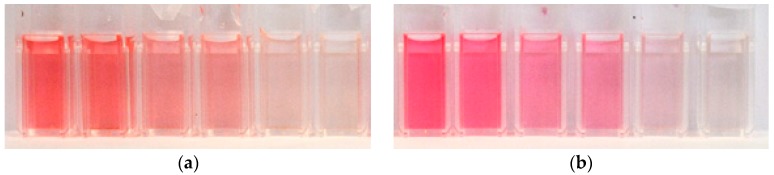
Dye extracts of pH = 1.00, 2.00, 2.89, 3.00, 4.00, and 5.00 from left to right, (**a**) without caffeine and (**b**) with caffeine.

**Figure 3:**
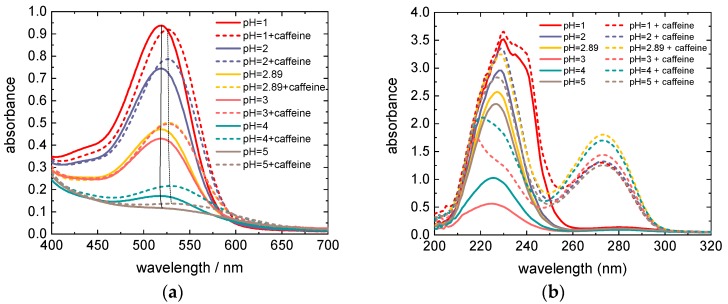
(**a**) UV/Vis spectra of dye solutions of different pH values, pure (solid lines) and copigmented with caffeine (dotted curves) with a caffeine to anthocyanin molar ratio of 229:1. The thin black solid and dotted lines connect the absorption maxima of anthocyanins of different pH values without and with caffeine, respectively. (**b**) UV region of the absorption spectra of dye extracts from subfigure (**a**) diluted by a factor 5.

This is clearly visible in Figure 2a which shows dye solutions of the same molar concentration of anthocyanins, but increasing pH values from left to right. Correspondingly, the height of the maximum at 520 nm in the absorption spectra is decreasing with increasing pH (see the full lines in Figure 3a; the dashed lines and Figure 3b will be discussed later). It must be mentioned that here the height of the maximum is proportional to the molar concentration of colored molecules (i.e., flavylium cations and quinoidal bases) and not to the molar concentration of total anthocyanin content in the solution. The samples with pH = 4 and pH = 5 are almost colorless, and the absorption maximum at 520 nm has vanished, indicating that in this pH range only a minor fraction of colored species is present in the dye solution. Assuming that each species adsorbs equally well on TiO_2_, at moderate acidic conditions also colorless molecules are adsorbed on the semiconductor_._ These molecules, since they are unable to absorb light, do not contribute to the energy conversion, leading to a decrease of the energy-conversion efficiency.

**Figure 4:**
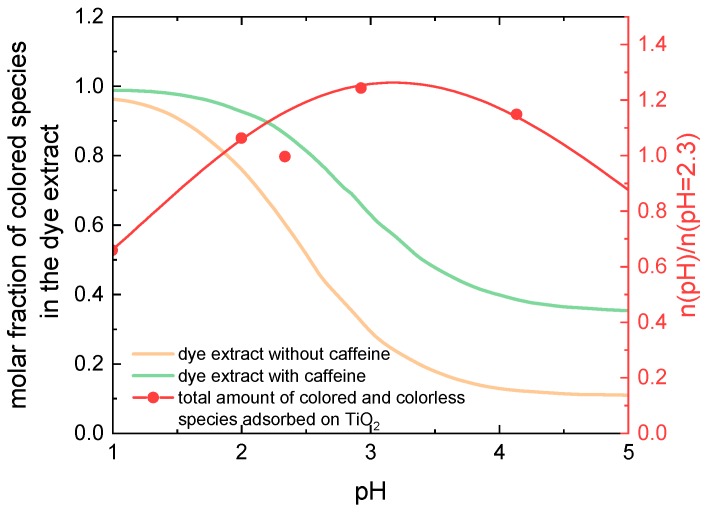
pH-dependence of molar fractions of colored species in the pure dye extract (orange line) and in the dye extract copigmented with caffeine (green line), obtained as the sum of molar fractions of flavylium cations and quinoidal bases in pure and copigmented dye, reprinted (adapted) with permission from Ref. [22] (Copyright, 2013, American Chemical Society); compared to the pH-dependence of the amount of dye adsorbed on TiO_2_ (red symbols and line), reprinted (adapted) with permission from Ref. [12] (Copyright, 2017, American Institute of Mathematical Sciences).

The orange curve in Figure 4 shows the pH dependence of the molar fraction of colored molecules in an anthocyanin solution, i.e., the sum of molar fractions of flavylium cations and quinoidal bases taken from Ref. [22]. Additionally, the red symbols and curve show a physical quantity which is proportional to the amount of all anthocyanins (sum of colored and colorless molecules) which at a given pH value are adsorbed on TiO_2_ (adopted from Ref. [12]). Comparing the two curves, we can see that at pH = 3, where the dye adsorption is most intensive, the molar fraction of colored molecules in the solution is only about 30%, opposite to over 90% at pH = 1. Correspondingly, at pH = 1, a much larger amount of colored molecules adsorbs on the TiO_2_ layer than at pH = 3. This explains the reduced energy-conversion efficiency at pH = 3, since the efficiency is proportional to the amount of colored molecules adsorbed on the TiO_2_ surface. Obviously, an increase of the molar fraction of colored molecules in the dye solution at pH = 3 would lead to an efficiency increase and probably to the shift of the pH of maximal efficiency from strongly acidic into the moderate acidic region.

The molar fraction of colored molecules in a moderate acidic anthocyanin solution can be increased, as it was mentioned in the introduction, by addition of a copigment (for example caffeine) to the dye [21,22,23] (compare Figure 2a,b). A copigment is a colorless substance which intensifies the dye color in the solution. Figure 4 shows the pH-dependence of the sum of molar fractions of flavylium cations and quinoidal bases taken from Ref. [22] in a 2 × 10^−5^ M anthocyanin solution (orange line) and in the same solution with added caffeine (0.08 M, green line). Obviously, under the influence of the copigment with a caffeine to anthocyanin molar ratio equal to 4000:1, in moderate acidic conditions (for example at pH = 3) the molar fraction of colored molecules is almost doubled compared to the solution without caffeine [22].

A rough estimate of the amount of colored species adsorbed on TiO_2_ can be obtained as a product of the molar fraction of colored species in the dye solution and the total amount of dye adsorbed on TiO_2_. Since the efficiency of the DSSC is proportional to the amount of adsorbed colored dye molecules, based on Figure 4, an increase of the efficiency for all pH ≥ 2 and the shift of the highest efficiency found in previous work for pH = 1.1 [12] into the pH region between 2 and 3 is expected.

**Figure 5:**
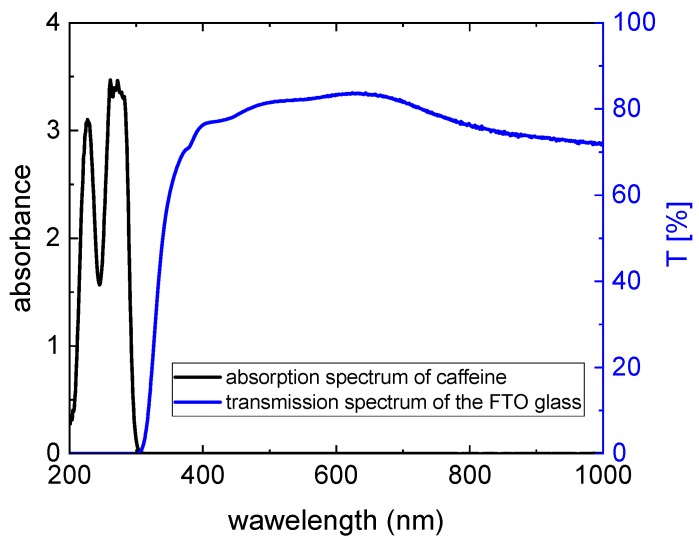
Absorption spectrum of caffeine (black curve) together with the transmission spectrum of FTO glasses used for preparation of DSSCs.

For the investigation of the influence of copigmentation on the DSSC properties, dye extracts with and without caffeine, with pH values varying between pH = 1 and pH = 5, were prepared as described in the section Materials and Methods. The molar concentration of anthocyanins in the dye extract was estimated from the UV/Vis the spectra of extracts with pH = 1 and pH = 4.5, in the same way as proposed in Ref. [25], using the following formula:

(1) $$c\left(\frac{\mathrm{mol}}{L}\right)\;=\;\left[{\left(A_{520}\;-{\;A}_{700}\right)}_{\mathrm{pH}\;=\;1.0}\;-\;{\left(A_{520}\;-{\;A}_{700}\right)}_{\mathrm{pH}\;=\;4.5}\right]\frac{DF}{Ɛ\;l}$$

where *DF* = 5 is the dilution factor and *l* = 1 cm is the path length of light through the solution. Since the exact chemical composition of the dye extract is unknown, the molar absorptivity of cyanidin-3-glucoside Ɛ = 26,900 L/(mol·cm) was used for the calculation of the dye concentration, as recommended in Ref. [25]. With absorbances determined at 520 nm and 700 nm, namely: (A_520_)_pH = 1.0_ = 0.937 and (A_700_)_pH = 1.0_ = 0.012 as well as (A_520_)_pH = 4.5_ = 0.135 and (A_700_)_pH = 4.5_ = 0.018, the molar concentration of anthocyanins *c* = 1.5 × 10^−4^ mol/L in the dye extracts is obtained. The molar concentration of anthocyanins is equal in all samples (all pH values) and is 7.5 times higher than in Ref. [22]. The high concentration is necessary to achieve a good adsorption on TiO_2_. The molar concentration of caffeine in dye extracts with the copigment equals c = 0.034 mol/L, yielding a caffeine to anthocyanin molar ratio of 229:1, which is much lower than in Ref. [22]. However, the copigmentation effect is expected to be well pronounced in our dye extracts, since according to Refs. [21,22,23] it appears already at a caffeine to anthocyanin molar ratio of 10:1. In Figure 2, which shows the 5× diluted pure (a) and copigmented (b) dye extracts with different pH values, the influence of the copigmentation on the dye color is clearly visible. The visible region of absorption spectra of these dye extracts is shown in Figure 3a. Although the molar concentration of anthocyanins is equal in all dye extracts, with increasing pH the color of extracts fades (see Figure 2), i.e., the absorption maximum decreases in pure as well as in copigmented extracts (see Figure 3a) as a result of decreasing molar fraction of colored anthocyanin molecules through stronger hydration of flavylium cations for increasing pH [21,22,23]. Compared to the spectra of pure anthocyanin solutions, the spectra of copigmented anthocyanins show a hyperchromic effect for pH > 1 and a bathochromic shift of the absorption maximum by 8–10 nm, depending on the pH value. The bathochromic effect can be explained by the shift of the deprotonation equilibrium towards the quinoidal base in the presence of caffeine, while the hyperchromic effect is a result of the shifted hydration equilibrium towards the flavylium form, whose concentration in the solution is increased at the expense of colorless hemiketals [22]. Figure 3b shows the UV region of the absorption spectra of dye solutions from Figure 3a diluted by a factor 5. It is obvious that the pure anthocyanins show an absorption maximum between 200 nm and 260 nm (see the solid lines), while the dye solutions with added caffeine exhibit an additional maximum between 250 nm and 300 nm. These latter maxima occur as the result of light absorption through caffeine. Compared to the maxima in the visible region, the absorption maxima in the UV region are significantly higher. However, they do not contribute to the energy conversion since the glass plates used for the preparation of DSSCs are not transparent for short-wavelength radiation (see Figure 5). Figure 5 shows the transmission spectrum of an FTO glass compared to the absorption spectrum of caffeine. Both spectra do not overlap; therefore, the energy-conversion efficiency of DSSCs is not affected by the absorption through caffeine. Since the absorption effects in the UV region are irrelevant for the energy conversion, they will not be discussed further.

**Figure 6:**
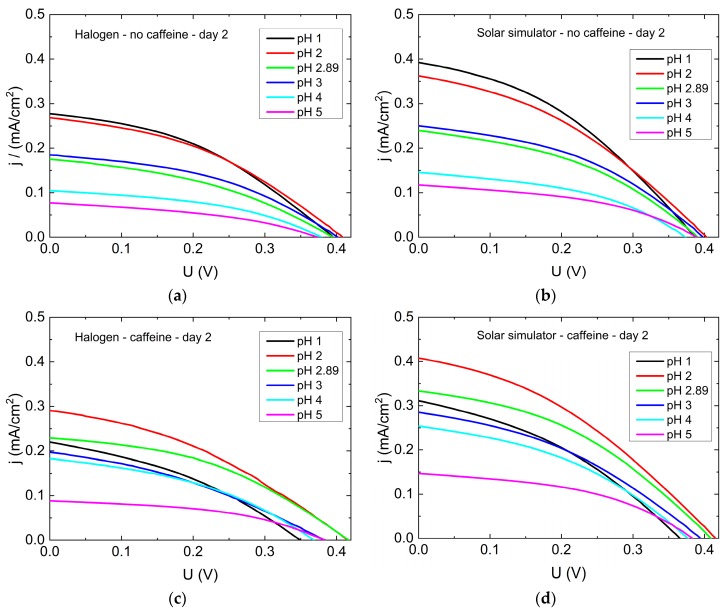
Current density versus voltage of cells prepared with different pH values: (**a**) without caffeine, measured with the halogen lamp; (**b**) without caffeine, measured with the solar simulator; (**c**) with caffeine, measured with the halogen lamp; (**d**) with caffeine, measured with the solar simulator.

Figure 6 shows exemplary results of current density-voltage (j-U) curves of DSSCs prepared without or with caffeine in the dye solutions (molar concentration of anthocyanins *c* = 1.5 × 10^−4^ mol/L). The measurements started on day 2 after preparation to enable a complete penetration of the dye into the whole DSSC, including the pores of the semiconductor. Without caffeine, all open-circuit voltages are similar, and the short-circuit currents are highest for the lowest pH value. All dye solutions with tailored pH values follow the expected trend of decreasing efficiency with increasing pH value. The untreated tea solution with pH = 2.89 shows a slightly lower current than expected in comparison with the other curves, which we cannot explain at the time. This finding will be examined further in the future.

For the j-U curves of the DSSCs with caffeine, the highest short-circuit currents can be found for pH = 2, as already expected due to the physical mechanism discussed in association with Figure 4. Here, the open-circuit voltages also differ visibly, with the highest values for pH = 2 and pH = 2.89 and the lowest ones for pH = 1. Nevertheless, the lowest currents and thus the smallest efficiencies can again be expected for pH = 5, according to these graphs. Generally, for both cell types (with and without caffeine) the short-circuit current and the open-circuit voltage are smaller than expected. However, the open-circuit voltage is comparable to that obtained by Ghann et al. [26], who built DSSCs with natural dyes and graphite coated counter electrodes. In a previous work, we obtained a similar open-circuit voltage for cells with textile based counter electrodes built from electrospun polyacrylonitrile nanofiber mat, which was coated by poly(3,4-ethylenedioxythiophene)- poly(styrenesulfonate) (PEDOT:PSS) and graphite [27]. For an improvement of DSSC characteristics further work on the other layers is necessary.

**Figure 7:**
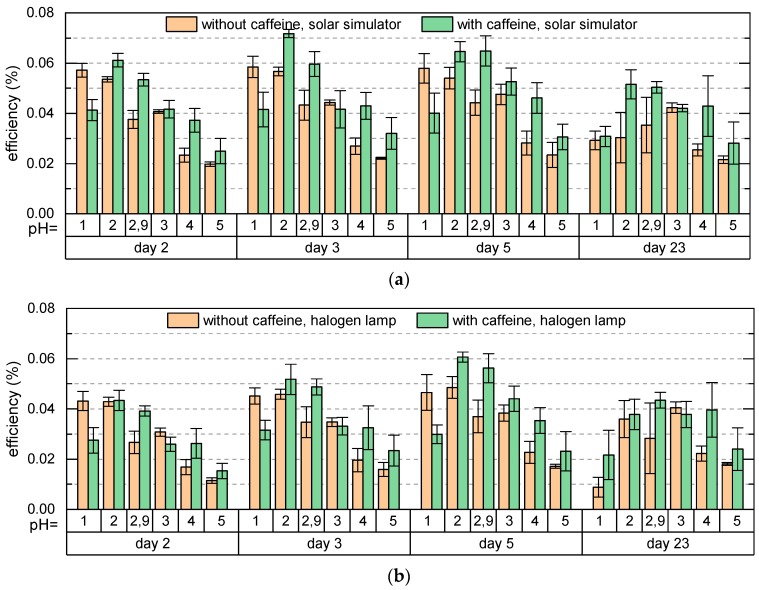
Efficiencies of DSSCs dyed with pure anthocyanin dye (orange bulks) and anthocyanin dye copigmented by caffeine, using a caffeine to anthocyanin molar ratio of 229:1 (green bulks), (**a**) measured with the solar simulator, (**b**) measured with the halogen lamp.

Figure 7 shows the efficiencies of DSSCs dyed with anthocyanins with and without copigments, illuminated by AM 1.5 G spectrum and by a halogen lamp of 3000 K color temperature (simulating ambient light) measured for several days. The efficiencies show a similar behavior under both illumination conditions; however, under the halogen lamp they are slightly lower than under the solar simulator. In agreement with the previous results [12], the efficiency of DSSCs dyed with pure anthocyanins is maximal for the lowest pH value of the dye extract (pH = 1), and decreases with increasing pH. The reason is the decreasing molar fraction of colored molecules in the dye solution with increasing pH (corresponding to the decrease of the absorption maxima in Figure 3a) and thus a decreased fraction of colored species adsorbed on the TiO_2_.

For the cells with copigmented dye, the efficiency is maximal for pH = 2 and decreases for increasing pH due to the same reason as for cells with pure dye. For pH ≥ 2, the efficiency of cells with caffeine is higher than the efficiency of cells with pure anthocyanins as a result of the hyperchromic effect in presence of caffeine (see Figure 3a). For pH = 1 we have an opposite situation. Here the efficiency of cells with pure dye is higher than the efficiency of cells with copigmented dye corresponding to the hypochromic effect observed for pH = 1 (see Figure 3a). Even though the maximum absorbance for dye with caffeine at pH = 1 is higher than at pH = 2, the efficiency of cells with pH = 2 is larger. We ascribe this to the better adsorption of the dye on the TiO_2_ surface at pH = 2 than at pH = 1. The increased efficiency occurs entirely due to the adsorption of anthocyanins which absorb in the region 400–600 nm and not due to added absorption effects at higher energies, since the glass plates used for preparation of DSSCs filter out the spectral region below 300 nm (see Figure 5). To resume, the shift of the maximal efficiency of cells with copigmented dye towards higher pH values and the increased efficiency of cells with copigmented dye compared to cells with pure dye agree with our prediction based on Figure 4 and justify the hypothesis that the copigmentation of an anthocyanin-dye extract by caffeine at moderate pH values increases the fraction of colored species adsorbed on TiO_2_, resulting in an increase of the efficiency. Besides the increased efficiencies, the shift of the maximal efficiency into the less acidic region is another advantage of using the copigmented dye, especially for the development of textile-based solar cells, which is our aim. The second highest efficiency was found for the original pH = 2.89. Two days after the cell preparation the efficiency of cells with caffeine for pH = 2.89 is slightly lower than the highest efficiency of cells with pure dye (pH = 1); however, it is much more stable. While during the measurement period of 23 days, the efficiency of cells with pure pH = 1 dye decreases by about 50%, the efficiency of cells with pH = 2.89 copigmented dye decreases only by about 16%, so that at the end of the measurements it is by about 66% higher than the efficiency of the cells with pure anthocyanins of pH = 1. This indicates that an anthocyanin dye extract with its original pH value copigmented by caffeine is a good choice for dyeing the DSSCs. The cells with pH = 4 were found to be most stable during the time. Here again, the cells with caffeine show higher efficiencies than the cells with pure dye.

**Figure 8:**
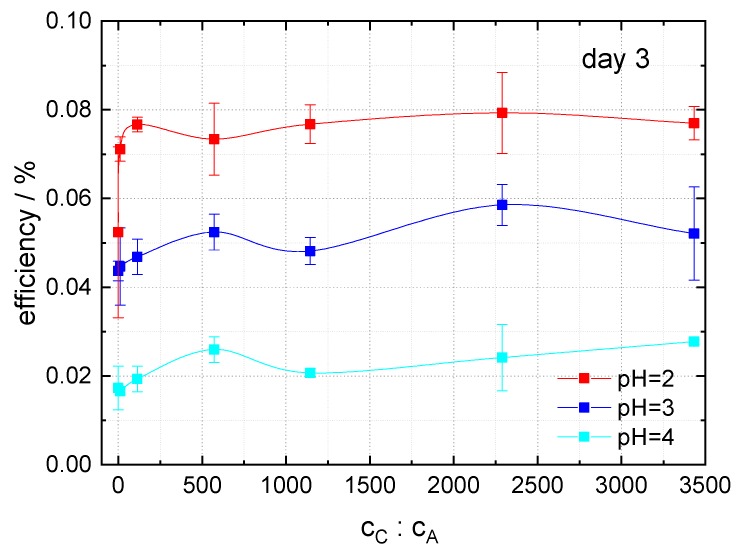
Dependence of the energy-conversion efficiencies of DSSCs dyed with copigmented dyes of pH = 2, 3, and 4 on the caffeine-to-anthocyanin molar ratio c_C_:c_A_. The measurements were carried out three days after the preparation of cells under AM 1.5 G illumination (solar simulator).

In order to investigate the influence of the caffeine to anthocyanin molar ratio in the dye extract on the energy-conversion efficiency of DSSCs, dye extracts with pH = 2, 3 and 4 were prepared, and the caffeine to anthocyanin molar ratio was varied between 0:1 and 3433:1. Here, the anthocyanin molar concentration was 10× lower than in previous examinations, in order to achieve higher copigment-to-dye molar ratios without exceeding the solubility limit of caffeine. The results, measured three days after the preparation of cells, are shown in Figure 8. For all three pH values, the efficiency increases after addition of small amounts of caffeine to the dye, achieving a maximum followed by a minimum and a further slight increase with increasing caffeine content in the solution. For high caffeine to anthocyanin molar ratios the efficiency is nearly constant. For pH = 2 and 3, the best results were found for a caffeine-to-anthocyanin ratio of 2289:1, whereas for pH = 4, the efficiency increases in the investigated region. 

Finally, Table 1 gives an overview of typical efficiencies gained with similar DSSCs. It should be mentioned that there is a tendency of miscalculating these values; thus in Table 1 the correct efficiencies, as calculated from the numbers given in the respective papers, are given together with the erroneously given efficiencies from the papers, if necessary. The second column mentions special treatments of one or more layers.

From Table 1 it is clearly visible that enhancing the dye alone will not lead to a strong increase of the efficiency. In the cells with efficiencies larger than ~0.1%, there is typically a platinum (Pt) catalyst used, which is not useful in the planned textile applications.

In most cases, only small cells are used, making current flow easier and increasing the homogeneity of the minimized areas, which is opposed to the idea of using DSSCs in large-scale textile fabrics, e.g., in textile architecture, and thus also not performed in our article. Several papers do not even mention the cell dimensions.

Finally, often Li-based and other unhealthy electrolytes are applied which is impossible in textile fabrics, as well as using ethanol or other inflammable or toxic solvents, while in the textile industry, finishing processes are in most cases water-based.

As this comparison shows, creating a relatively highly efficient solar cell with anthocyanins as the dye necessitates modification of all layers. Since platinum as catalyst, ethanol as solvent for the dye or Li based electrolytes cannot be used in textile-based DSSCs, other approaches are necessary to develop DSSCs based on non-toxic, non-inflammable materials further, e.g., by using electrospun electrodes [27]. Here, however, the focus is on enhancing the dye without using a strongly acidic regime, while all other parameters are standard materials to enable comparison with other groups.

## 4. Conclusions

In this paper, the effect of copigmentation of anthocyanin dyes on the energy-conversion efficiency of DSSCs was investigated. It was found that a copigmentation with caffeine increases the efficiency and leads to a shift of the maximal efficiency from the strongly acidic region into the moderately acidic region. This allows dyeing of the cells with dyes with their original pH value. Furthermore, the cells with copigmented dyes were found to be more stable than the cells with pure anthocyanins, i.e. they showed a smaller drop of efficiency during the observation period.

## Figures and Tables

**Table 1:** Overview of typical efficiencies η of DSSCs prepared with anthocyanins.

Authors	Deviations from Our Setup	η (Correct)	η (Acc. to Paper)	Ref.
Alhamed et al.	Pt counter electrode, Li electrolyte, cell area not given	0.075%	1.50%	[10]
Al-Bhat’hi et al.	Ethanol as solvent, solid-state electrolyte, cell area not given	0.036–0.15%	0.36–1.5%	[11]
Juhász Junger et al.		0.06%		[12]
Lucioli et al.	Ethanol as solvent, two TiO_2_ layers, Pt counter electrode, cell area 25 mm²	0.19%	0.31%	[13]
Juhász Junger et al.		0.089%		[15]
Hölscher et al.		0.03%		[28]
Zhou et al.	Ethanol as solvent, concentrated filtrates, only 0.2 cm² cell area, Pt counter electrode, Li electrolyte	0.24%		[29]
Li et al.	Sputtered Pt nanocluster photocathode, Li electrolyte, cell area not mentioned	2.3%		[30]
Gokilamani et al.	Nano-crystalline TiO_2_, concentrated dye filtrates, Li electrolyte, cell area only 0.25 cm²	0.73%		[31]
Wongcharee et al.	Pt catalyst, cell area not mentioned	0.05–0.37%		[32]
Senthil et al.	Cell areas only 0.1–0.25 cm²	0.00021%	0.505%	[33]
Hao et al.	Ethanol as solvent, concentrated filtrates, only 1 cm² cell area, Pt counter electrode	0.163–0.327%	-	[34]
This paper	Cell area 6 cm²	0.08%		
